# Impact of resection margin status on survival in advanced N stage pancreatic cancer – a multi-institutional analysis

**DOI:** 10.1007/s00423-021-02138-4

**Published:** 2021-03-13

**Authors:** Christian Teske, Richard Stimpel, Marius Distler, Susanne Merkel, Robert Grützmann, Louisa Bolm, Ulrich Wellner, Tobias Keck, Daniela E. Aust, Jürgen Weitz, Thilo Welsch

**Affiliations:** 1 Department of Visceral, Thoracic and Vascular Surgery, University Hospital and Faculty of Medicine Carl Gustav Carus, Technische Universität Dresden, Dresden, Germany; 2 Department of General and Visceral Surgery, Friedrich Alexander University, Erlangen, Germany; 3 Department of Surgery, University Medical Centre Schleswig-Holstein, Campus Lübeck, Lübeck, Germany; 4 Institute of Pathology, University Hospital Carl Gustav Carus, Dresden, Germany

**Keywords:** PDAC, Resection status, Lymph node, Pancreatectomy, Survival

## Abstract

**Background:**

The present study aimed to examine the impact of microscopically tumour-infiltrated resection margins (R1) in pancreatic ductal adenocarcinoma (PDAC) patients with advanced lymphonodular metastasis (pN1–pN2) on overall survival (OS).

**Methods:**

This retrospective, multi-institutional analysis included patients undergoing surgical resection for PDAC at three tertiary university centres between 2005 and 2018. Subcohorts of patients with lymph node status pN0–N2 were stratified according to the histopathological resection status using Kaplan-Meier survival analysis.

**Results:**

The OS of the entire cohort (*n* = 620) correlated inversely with the pN status (26 [pN0], 18 [pN1], 11.8 [pN2] months, *P* < 0.001) and R status (21.7 [R0], 12.5 [R1] months, *P* < 0.001). However, there was no statistically significant OS difference between R0 versus R1 in cases with advanced lymphonodular metastases: 19.6 months (95% CI: 17.4–20.9) versus 13.6 months (95% CI: 10.7–18.0) for pN1 stage and 13.7 months (95% CI: 10.7–18.9) versus 10.1 months (95% CI: 7.9–19.1) for pN2, respectively. Accordingly, N stage–dependent Cox regression analysis revealed that R status was a prognostic factor in pN0 cases only. Furthermore, there was no significant survival disadvantage for patients with R0 resection but circumferential resection margin invasion (≤ 1 mm; CRM+; 10.7 months) versus CRM-negative (13.7 months) cases in pN2 stages (*P* = 0.5).

**Conclusions:**

An R1 resection is not associated with worse OS in pN2 cases. If there is evidence of advanced lymph node metastasis and a re-resection due to an R1 situation (e.g. at venous or arterial vessels) may substantially increase the perioperative risk, margin clearance in order to reach local control might be avoided with respect to the OS.

**Supplementary Information:**

The online version contains supplementary material available at 10.1007/s00423-021-02138-4.

## Introduction

Current multimodal treatment strategies of pancreatic ductal adenocarcinoma (PDAC) have resulted in a significant survival benefit for patients with localized or even locally advanced tumours. High-impact randomized results of surgical resection followed by modern adjuvant multi-agent chemotherapy for localized PDAC have shown an overall survival of 28 to 54 months (using modified FOLFIRINOX) [1, 2]. Of note, the best outcome was achieved within the R0 (microscopically tumour-free resection margin) subgroup of patients compared with cases in which the resection margins harboured microscopic tumour invasion (R1) [2]. Intraoperative surgical clearance of the resection margin is an ongoing matter of debate [3]. Radical R0 resection, especially in vulnerable and critical anatomic structures (e.g. the portal vein, arterial vessels, or the pancreatic neck margin), may significantly increase the intra- and perioperative risk for the patient and result in attempts of re-resection of the portal vein anastomosis, arterial reconstruction or completion pancreatectomy.

Besides R status, regional lymph node metastases (pN) are a strong prognostic survival determinant after resection of PDAC. This formed the basis for the recent revision of the N classification [4, 5]. In accordance with the 8^th^ edition of the American Joint Committee on Cancer (AJCC) guidelines, PDAC is now classified based on the total number of infiltrated lymph nodes as pN1 (1–3 lymph nodes) and pN2 (>3 lymph nodes) [6]. Extended lymphadenectomy has still not shown any significant survival advantage compared with standard lymph node dissection so far; however, selected patients may benefit from this approach [7, 8].

Therefore, although there is evidence that both the R and pN stage are significant prognostic factors, the question remains of whether local control (R0 status) matters in advanced lymphonodular positive disease (e.g. pN2 according to the new N classification). In gastric cancer surgery, for instance, a positive proximal resection margin is associated with advanced N stage; however, it is not independently associated with survival in these cases and recurrence is mainly systemic [9, 10].

The present study therefore aimed to examine the clinical impact of the R status during PDAC resections in a multimodal treatment era including the use of neoadjuvant treatment on patients’ survival with respect to the different lymph node stages (pN0–N2).

## Methods

### Study design, patients, and pathology reporting

The present study was designed as a retrospective German multicentre analysis. Data were collected from 3 different pancreatic cancer databases of the following tertiary university centres: Department of Visceral, Thoracic and Vascular Surgery, University Hospital Dresden (2005–2017), Department xof Surgery, University Hospital Erlangen (2010–2018), and Department of Surgery, University Medical Centre Schleswig-Holstein, Campus Lübeck (2013–2018). All patients undergoing surgical resection for PDAC within the indicated timeframes were retrospectively analyzed. The surgical procedures included pylorus-preserving (PPPD) or classic (cPD) pancreatoduodenectomy, total pancreatectomy (TP), distal pancreatectomy (DP), completion pancreatectomy, and the Appleby procedure (Appleby). During the study period, the three participating centres had increasingly utilized neoadjuvant chemotherapy protocols (e.g. FOLFIRINOX) for borderline resectable cases according to the international classification [11]. Tumours with unilateral narrowing (superior mesenteric or portal vein contact [SMV] of less than 180°) of the SMV/portal vein were usually classified resectable and resected upfront. Intraoperative resection margins of the pancreatic neck and bile duct were routinely sent for frozen section analysis. A re-resection for margin clearance was aimed at in cases of tumour cell infiltration. Pathology reports were retrospectively screened and the pN category was adjusted to pN1 (1–3 infiltrated lymph nodes) and pN2 (>3 infiltrated lymph nodes) according to the latest 8^th^ AJCC classification. All subjects were stratified into three subgroups with respect to pN category and resection status (R0 or R1) as follows: pN0-R0/1, pN1-R0/1, and pN2-R0/1. R0 status was defined as microscopic absence of tumour cells at the resection margin in all centres. Beginning in the year 2010, R0 resections were subdivided into CRM+ (tumour cells within 1 mm of the circumferential resection margin) and CRM− (no tumour cells within 1 mm of the CRM) in all three participating centres according to the national consensus guidelines. The CRM classification was considered for subsets of patients. The R0 CRM+ status corresponds to the redefined R1 resection margin involvement introduced by Verbeke et al. [12]. In addition, para-aortic lymph nodes of the Ln16b1 station (PALN) were analyzed separately if they were unequivocally labelled. Kaplan-Meier survival curves were generated with respect to the pN and R status.

Postoperative complications were classified according to the Clavien-Dindo Classification [13]; postoperative pancreatic fistula (POPF) was categorized into biochemical leakage (no fistula) and clinically relevant fistulae (Grade B and C) [14].

Follow-up was defined as the time between the index operation and the last contact with a physician, which was obtained from the respective hospital information system or by contacting the patient’s general practitioner.

### Statistical analysis

The open source software package R Studio was used (Version 1.2.1335; https://rstudio.com/products/rstudio/) for the statistical calculations and creating data plots. Data are presented as median with interquartile range (IQR) or 95% confidence interval (95% CI) unless otherwise indicated. For comparing the patient subgroups, the *t*-test (continuous variables, normal distribution), Wilcoxon-Mann-Whitney (continuous variables, not normal distribution), or chi-square tests (categorical variables) were used. The statistical significance level was set a priori to *P* = 0.05 for all calculations. Kaplan-Meier survival curves for OS were generated considering the indicated parameters. OS was defined as the time between index operation and death or last appointment (censored). The logrank test was performed for testing differences between survival curves. For R-dependent survival analysis, Rx (unclear resection margin) and R2 (macroscopic residual tumour) resection status were excluded.

Univariate Cox proportional model analysis was calculated including the indicated variables. The lymph node ratio (LNR) was calculated as the quotient between infiltrated and resected lymph nodes. Statistically significant parameters in the univariate model were included in the multivariate analysis model. The hazard ratio was calculated and is shown.

## Results

### Description of the study cohort and overall survival

**Fig. 1:**
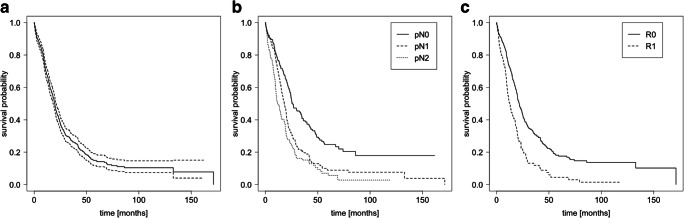
Survival analysis with respect to lymph node and resection status. Kaplan-Meier survival curves of all patients (*n* = 620) were calculated and plotted for overall survival (OS). **a** The complete study cohort was analyzed and a 95% CI calculated (displayed as dashed lines). **b**, **c** pN status and resection status–dependent OS was examined and shown as indicated. A logrank test was performed and statistically significant differences were obtained (*P* < 0.001). R2 and Rx resection classifications are not shown

In total, 620 patients were included in the retrospective multicentre analysis; the median follow-up was 16.5 (IQR: 8.5–26.8) months. The median age was 66.9 (IQR: 61–74) years and 312 patients (50.3%) were male (Table 1). The most common types of resections were pancreatic head resections (73.5%), TPs (10.8%), and DPs (15.2%). The median overall survival (OS) was 19.7 (95% CI: 18–20.9) months (Fig. 1a). Application of the revised TNM classification for pathohistological reporting resulted in 233 cases with no lymph node metastasis (pN0, 37.6%), 241 cases with pN1 stage (38.9%), and 146 cases with pN2 (23.5%) stage [6]. Considering the entire cohort, pN0 status was significantly associated with the longest median OS of 26 (95% CI: 24.1–34.7) months, followed by 18 months for the pN1 stage (95% CI: 16.1–20.1 months) and 11.8 months for the pN2 stage (95% CI: 10.1–16.1 months; *P* < 0.001; Fig. 1b).

**Table 1:** Patient and operative characteristics of the entire study population

Variable	Value	%
Patients (*n*)	620	100
Median age (years) [IQR]	66.9 [61–74]	-
Male sex (*n*)	312	50.3
Median follow-up (months) [IQR]	16.5 [8.5–26.8]	-
Median CEA (μg/l) [IQR]	2.4 [1.4–4.2]	-
Median CA19-9 (U/ml) [IQR]	130 [33.2–521]	-
Operation (*n*)		
-PPPD -cPD	340 116	54.8 18.7
-TP	67	10.8
-DP	95	15.3
-Completion pancreatectomy	2	0.3
Neoadjuvant treatment (*n*)	95	15.3
-CTx	20	
-RCTx	47	
-Not specified	28	
Adjuvant treatment (*n*)	329	53.1
-CTx	238	
-Gemcitabine based	201	
-RCTx	13	
-Not specified	78	
PV resection (*n*)	207	33.4
Arterial resection (*n*)	43	6.9
Median blood loss (ml) [IQR]	625 [400–1125]	-

*cPD*, classical pancreatoduodenectomy; *CTx*, chemotherapy; *DP*, distal pancreatectomy; *IQR*, interquartile range; *PPPD*, pylorus-preserving pancreatoduodenectomy; *PV*, portal vein; *RCTx*, chemoradiation; *TP*, total pancreatectomy

Most of the patients were resected with microscopically tumour-free resection margins (R0, *n* = 469, 75.6%). Within this R0 subgroup, the CRM classification was available for 379 cases: 191 (40.7%) cases did not show any tumour infiltration within 1 mm of the resection margins (CRM−), whereas tumour cells were found within 1 mm of the margin in 188 cases (CRM+; 40.1%). In 90 patients, a retrospective analysis of the microscopic specimen and reclassification according to CRM status was not possible because they were analyzed before the implementation of the CRM classification. An R1 resection status was found in 124 cases (20%). The majority of these cases (*n* = 42, 33.9%) had remaining PDAC cells at the pancreatic resection margin, followed by 28 patients (22.6%) having residual tumour within the medial (vessel) margin and 25 patients with a retroperitoneal R1 situation (20.2%) (Table 2). R status–dependent analysis of the patients’ OS demonstrated a significant difference with 21.7 (95% CI: 19.0–24.2) months and 12.5 (95% CI: 10.2–16.9) months for R0 and R1 resected patients, respectively (Fig. 1c).

**Table 2:** Pathohistological tumour staging (entire cohort, *n* = 620)

Stage and margin status	*n* (%)
T	
pT1	48 (7.7)
pT2	115 (18.5)
pT3	424 (68.4)
pT4	17 (2.7)
N	
pN0	233 (37.6)
pN1	241 (38.9)
pN2	146 (23.5)
M	
pM0	561 (90.5)
pM1	56 (9.0)
R status	
R0	469 (75.6)
R0, CRM−	191 (40.7)
R0, CRM+	188 (40.1)
R1	124 (20.0)
R2	5 (0.8)
Rx	22 (3.5)

pTx, pNx, and pMx are not demonstrated

### Lymph node status–dependent survival analysis

**Fig. 2:**
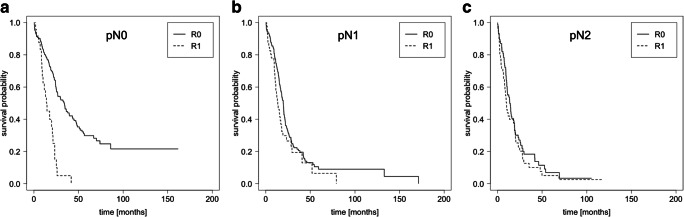
Resection status–dependent survival analysis with respect to lymph node classification. Kaplan-Meier survival curves were calculated for the R0 and R1 resection margin status with respect to lymph node classification pN0 (**a**; *n* = 221: R0 [196 patients] vs. R1 [25 patients]), pN1 (**b**; *n* = 232: R0 [184 patients] vs. R1 [48 patients]) and pN2 (**c**; *n* = 140: R0 [89 patients] vs. R1 [51 patients]). Using the logrank test, a significant OS difference was only obtained in the pN0 cohort (*P* < 0.001). R2 and Rx resection status classifications are not shown

Next, subgroup OS analyses were performed stratified by lymph node metastasis stage. The entire study cohort consisted of 233 (37.6%) patients with pN0 stage. Subdivision of the pN0 subgroup according to R status revealed a significant median survival advantage for R0 resected patients: 33.9 (R0; 95% CI: 25.3–43.6) months compared with 14.37 (R1; 95% CI: 10.2–23.4) months (*P* < 0.001; Fig. 2a). There was still a trend towards a significant survival advantage for patients with limited lymph node positive disease (pN1) and R0 resection (19.6 months [95% CI: 17.4–20.9]) compared with R1 resection (13.6 months [95% CI: 10.7–18.0]) (*P* = 0.06) (Fig. 2b).

However, patients with advanced lymph node metastasis stage (pN2) had a comparable median OS of 13.7 months for R0-patients (95% CI: 10.7–18.9) versus 10.1 months for R1-patients (95% CI: 7.9–19.1) (*P* = 0.3; Fig. 2c). The same analysis was performed with a homogenous subcohort of patients having received adjuvant chemotherapy and no neoadjuvant treatment (*n* = 283). Likewise, there was no survival benefit for patients with pN2 status (*n* = 73) and R0 resection (17.7 months [95% CI: 13.6–26.0]) compared with R1 cases (19.7 months [95% CI: 10.1–28.1]) (*P* = 0.8; Supplementary data, Fig. S1).

We further aimed to investigate procedure-specific analysis for tumours localized in the pancreatic head or tail. Patients who underwent resections for head and body tumours (cPD, PPPD, or TP) showed a significant OS benefit of 34.4 months for R0 resections compared with 14.4 months for R1 resections in the pN0 subcohort (*P* < 0.001; *n* = 179). However, in the pN2 group (*n* = 132), no significant survival benefit could be detected (R0: 13.7 months vs. R1: 9.6 months, *P* = 0.3).

There were only 94 patients with DP for tumours localized in the pancreatic tail (pN0 = 42 cases, pN1 = 43 cases, pN2 = 9 cases). In this subgroup, a median OS benefit was observed for patients with R0 resections compared with R1 resected patients (R0: 24.2 months vs. R1: 17.7 months, *P* = 0.03). A subanalysis for pN2 patients after DP was not possible because there was only 1 patient with a R1 resection in this subgroup.

Recently, para-aortic lymph node station Ln16b1 (PALN) has re-emerged in the discussion of standard lymphadenectomy for PDAC [8, 15]. Tumour invasion into this lymph node station is considered a distant metastasis. Furthermore, it is also an indicator of more advanced lymph node spread and aggressive tumour biology. Therefore, PALN metastases seem to clinically bridge the tumour spread from local (pN2) to distant metastasis (pM1). Currently, there is no consensus recommendation that these lymph nodes should be part of a standard oncologic lymphadenectomy. The present study cohort included 156 patients, where a PALN resection could be traced and analyzed based on the separately labelled lymph nodes in this area. Twenty-four (15.4%) of these cases displayed PALN tumour metastases (PALN+). PALN+ patients had a significantly worse OS of 8.5 (95% CI: 3.2–12.7) months compared with 19.6 months in PALN− patients (95% CI: 17.8–27; *P* < 0.001). The percentage of pN2 stages within the PALN+ patients was 63.0%. Subdivision according to R status was not applicable because of the low number of PALN+ cases with R1 status (*n* = 4).

**Fig. 3:**
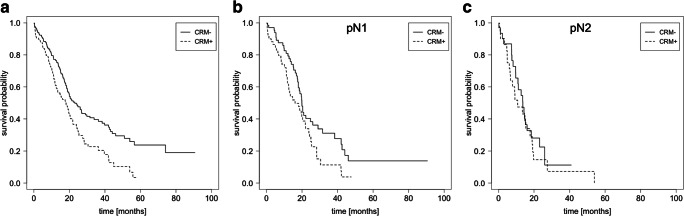
Survival analysis stratified by circumferential margin status and lymph node stage. Kaplan-Meier survival curves were calculated for the circumferential resection margin (CRM) status in R0 resected patients. With respect to the lymph node status, survival curves are shown for all (**a**; *n* = 313: CRM− [185 patients] vs. CRM+ [128 patients]), pN1 (**b**; *n* = 127: CRM− [67 patients] vs. CRM+ [60 patients]), and pN2 (**c**; *n* = 64: CRM− [32 patients] vs. CRM+ [32 patients]) patients. Significant survival differences were obtained for all (*P* < 0.001) and pN1 (*P* = 0.03) patients only. Patients with pN0 lymph node status are not shown

We further investigated whether CRM invasion impacts OS within the pN0, pN1, and pN2 subgroups because CRM reporting (considered standard according to German pancreatic cancer consensus guidelines) enables comparison of the traditional and redefined resection margin examination [12]. In the subset of cases with CRM reporting, the R0 cases were subdivided into 191 CRM− and 188 CRM+ cases. The median OS was significantly longer in the CRM− (23.1 months; 95% CI: 19–31.5) subgroup versus the CRM+ subgroup (18.1 months; 95% CI: 13–21.9; *P* < 0.001; Fig. 3a). Likewise, the median OS remained significantly improved for CRM− patients with pN1 stage (pN1R0 CRM−) compared with the pN1R0 CRM+ subgroup (19.9 months [95% CI: 18–29] versus 16.1 months [95% CI: 12.5–23.9]) (*P* = 0.03; Fig. 3b). In contrast, a tumour-free CRM was no longer associated with a survival advantage in extended lymph node metastasis (pN2): 13.7 months (CRM−; 95% CI: 10.9– 23.1) versus 10.7 months (CRM+; 95% CI: 6.7–19.2); *P* = 0.5; Fig. 3c).

### Characterization and subgroup analysis of patients with lymph node metastasis

In order to determine whether other confounder variables may influence OS between the R0 and R1 subcohorts, all cases with lymph node metastasis (pN+, i.e. pN1 and pN2 stage) were stratified according to R0 or R1 resection status (N+R0; N+R1) for descriptive analysis. The R0 rate in this subgroup was 73.4% (*n* = 273), including 137 (50.2%) CRM+ and 102 (37.4%) CRM− cases. When comparing the R0 and R1 subgroups, a significant difference was seen in the median preoperative value of tumour marker CA19-9 with 144 U/ml (IQR: 43.4–484.5 U/ml) and 252.4 U/ml (IQR: 53.8–929.8 U/ml) in pN+R0 and pN+R1, respectively (*P* < 0.001). In addition, the number of TPs (*n* = 22, 8.1%) was significantly higher in the R1 resection subgroup (*P* = 0.04). Other perioperative characteristics of the two subgroups were not significantly different (Supplementary data, Tab. S1).

### Prognostic survival determinants

Portal vein and arterial resection, complications ≥ grade 3, haemoglobin ≤7 mmol/l, lymph node ratio (LNR), and the lymph node status pN were found to be independent negative prognostic OS factors in the entire patient cohort (Table 3). Adjuvant systemic treatment was associated with a longer OS survival rate. The likelihood ratio for the calculated multivariate analysis revealed a comprehensive model (*P* < 0.001). Moreover, the pN status–dependent univariate Cox analysis showed R1 resection to be a significant prognostic factor for OS in lymph node negative patients only (*P* < 0.001; Supplementary data, Tab. S2).

**Table 3:** Uni- and multivariate Cox regression analysis for prognostic survival factors

	Multivariate		Univariate	
Variable	*p*-value	Hazard ratio	*p*-value	Hazard ratio
Age			0.105	1.01
Male sex			0.694	1.04
Stenosis of bile duct			0.853	1.02
Neoadjuvant therapy			0.379	1.12
Adjuvant therapy	**<0.001**	0.56	**<0.001**	0.62
PV resection	**<0.001**	1.44	**<0.001**	1.70
Arterial resection	**0.028**	1.48	**<0.001**	1.96
Complications ≥ grade 3	**<0.001**	1.88	**<0.001**	1.85
POPF (grade B-C)			0.151	1.30
Haemoglobin ≤ 7 mmol/l	**0.039**	1.28	**0.007**	1.36
LNR^*^	0.143	2.00	**<0.001**	5.57
Lymph node status pN	**0.002**	1.36	**<0.001**	1.52
Resection status				
R0	0.113	0.71	**<0.001**	0.55
R1	0.644	1.11	**<0.001**	1.85

*LNR*, lymph node ratio; *POPF*, postoperative pancreatic fistula; *PV*, portal vein

^*^Number of resected lymph nodes [median (IQR)]: all centres: 17 (IQR 13–23); centre #1: 17 (IQR 13–22.5); centre #2: 20 (IQR 14–25); centre #3: 15 (10–20)

Significant values are shown in bold

### Impact of neoadjuvant treatment

The present study also examined the impact of neoadjuvant treatment on OS with respect to lymph node stage because neoadjuvant chemotherapy addresses systemic tumour spread preoperatively (Supplementary data, Tab. S3 and Fig. S2). Compared with the entire study cohort, patients receiving neoadjuvant treatment (Neoadj+, *n* = 95) showed no survival benefit (median OS: 19.8 months [Neoadj−] versus 19.0 months [Neoadj+]; *P* = 0.4). These data have to be interpreted with caution because the majority of Neoadj+ patients had locally advanced tumours. Nevertheless, 83.2% of these patients undergoing neoadjuvant treatment were resected without any tumour cell infiltration of the resection border (R0, *n* = 79) whereas only 7 patients (7.4%) were classified R1. The rate of pN1 and pN2 patients within the Neoadj+ cases was 30.5% and 12.6%, respectively, which is lower than in patients without neoadjuvant treatment (38.9% [pN1]; 23.5% [pN2]). Considering the introduction of neoadjuvant chemotherapy protocols (e.g. FOLFIRINOX) beginning in 2011, a subcohort including only patients treated between 2011 and 2018 was analyzed (*n* = 473). In this subcohort, the R1 status was still only a significant survival determinant in pN0 cases (*P* < 0.001; Supplementary data, Tab. S4).

## Discussion and conclusion

In the present study, resection margin status seems to lose prognostic relevance in patients with advanced lymphatic tumour cell spread. This is relevant because the analyzed data when obtained from an era when multimodal treatment with neo- or adjuvant (poly-) chemotherapy for PDAC was standard, and advanced lymph node metastasis stages (pN2) according to current guidelines were considered. Very recently, the 8^th^ edition of the AJCC manual for cancer staging was published, which includes a new TNM classification system for pancreatic cancer [6]. The former N1 stage was subdivided into the N1 (1–3 positive lymph nodes) and N2 (>3 positive lymph nodes) stages.

In patients with positive resection margins (R1), Morales-Oyarvide and colleagues demonstrated that lymph node metastases have a weaker prognostic value for the patients’ disease-free and overall survival [16]. More importantly, several recent studies point to the fact that PDAC recurrence patterns (time or localization) are similar between R1 and R0 groups, especially in patients with N1 disease (7^th^ edition AJCC), and that lymph node status was predictive for time to recurrence, but not location of recurrence [17, 18]. The OS of patients with distant or local recurrence was not significantly different, which confirms that PDAC behaves as a systemic disease [19]. It is accepted that R1 resection provides a survival advantage over palliative treatment and R0 resection offers improved survival compared with R1 resections when all cases (including lymph node negative and positive patients) are considered [20]. Our group also found that resection margin clearance at the pancreatic neck margin using frozen section analysis leads to improved survival if all lymph node stages are included in the analysis [3]. In contrast, advanced lymph node metastasis stages probably reflect more advanced systemic tumour burden, which determines the further course and prognosis of the disease. At least in pN2 stages, systemic lymphonodular spread seems to outweigh the survival difference between local R0 and R1 margins, because the presented data revealed no significant difference in OS rate between R0 and R1 status in pN2 staged patients. However, our retrospective data set did not qualify for a differentiated recurrence pattern analysis in pN0, pN1, and pN2 stages and recent studies on the recurrence patterns after PDAC resection did not consider the advanced pN2 stages separately [17, 18, 21].

Given the heterogeneity of the pathological assessment of resection margins, the circumferential resection margin (CRM) concept was incorporated into national treatment guidelines several years ago [12, 22]. Therefore, an R0 resection is further classified as R0 CRM− (R0 wide) if microscopic tumour cells are found more than 1 mm from the resection margin. CRM+ cases (R0 narrow) show tumour cells within 1 mm of the resection margin but not within the margin itself. It has been shown that this concept comprehensively reflects the OS rate of PDAC patients [23]. To date, the classification of CRM status is still a matter of debate and no international consensus has been reached so far [24]. The CRM concept at least allows fine discrimination of the resection margin status or tumour cell density at the tumour margins. Patients with tumour cells within 1 mm from the resection margin (R0 CRM+) have a worse prognosis compared with R0 CRM− patients in pN0 or pN1 stages. Here again, no significant improvement in OS was observed for pN2 patients (*P* = 0.5).

Surgical margin clearance during pancreatic resection can be demanding and is associated with increased intraoperative risk. As more and more patients are explored with locally advanced tumours and involvement of the portal/superior mesenteric vein or visceral arteries, intraoperative frozen section analysis is a routine procedure for intraoperative assessment of surgical margin clearance at these critical vascular structures. This may be the case if the portal vein anastomosis is accomplished and the final venous resection margins (e.g. analyzed by frozen section) at the vein or at the visceral arteries display tumour cells. Both re-resection of the vein (e.g. superior mesenteric vein close to the mesenteric root) with subsequent re-anastomosis of the portal vein and arterial resection with reconstruction in the case of R1 situation at the hepatic or superior mesenteric artery can substantially increase the intra- and postoperative risk for the patient. This can also be true, if a re-resection of a R1-pancreatic neck margin frozen section requires a total pancreatectomy and there is evidence of advanced lymph node metastasis. Some centres even perform a completion pancreatectomy if an arterial resection is performed. The present data has clinical implications. According to the present data, attempts to radically clear these critical resection margins can be avoided if intra- or postoperative signs of advanced lymph node metastasis are evident or probable (e.g. by frozen section analysis of suspected lymph nodes or PALN). The presented data on PALN metastasis demonstrate that intraoperative evidence of PALN metastasis may be such an indicator for advanced lymph node metastasis (pN2).

In addition, adjuvant (chemo)radiation is recommended by some US centres after margin positive (R1) resection in lymph node positive patients [25]. The present results indicate that potential effects of adjuvant (chemo)radiation (versus modern systemic treatment alone) on disease-free or overall survival after R1 resection may be recapitulated with respect to the novel N-stages (pN1 and pN2). Several trials including the randomized ESPAC trials have shown that adjuvant chemotherapy is critical for control of the systemic burden of this disease. Only 38.4% (*n* = 238) of the entire cohort in the present study received a documented adjuvant chemotherapy, additional 12.6% of the patients (*n* = 78) received adjuvant treatment of unknown protocol (the data could not be obtained because of the retrospective multicentre design). The fact that almost half of the patients did not receive adjuvant chemotherapy is oncologically suboptimal and multifactorial (patient fitness, denial, etc.). The participating centres agree in the need for an increased rate of adjuvant treatment.

In order to increase the R0 resection rate, neoadjuvant therapy in borderline or non-resectable PDAC patients is at the centre of current clinical research. The latest data demonstrated a benefit in OS as well as R0 resection rates compared with upfront surgery. Moreover, neoadjuvantly treated patients showed less lymph node infiltration after surgical resection [26–30]. This further highlights the importance of achieving local tumour control (R0) during resection following neoadjuvant chemotherapy. The present data also demonstrate a reduction of lymph node metastasis after neoadjuvant treatment. This can be interpreted as a partial response to the neoadjuvant chemotherapy. Thus, local control in neoadjuvantly treated patients probably becomes more critical (less proportion of pN2 stages) and special attempts should be made to achieve R0 resections (including vascular reconstruction) in this patient subgroup for optimal outcome.

In conclusion, margin status (local control) seems not to be associated with OS in patients with advanced lymph node spread. A simultaneous or postoperative surgical re-resection in an R1 situation may be avoided for oncological reasons if there is evidence of advanced lymph node metastasis and the re-resection is associated with a substantially increased perioperative risk. These patients should be evaluated for modern adjuvant systemic treatment.

## Supplementary Information

ESM 1 (DOCX 27 kb)

ESM 2 (PNG 371 kb)

High resolution image (TIFF 4528 kb)

ESM 3 (PNG 253 kb)

High resolution image (TIFF 3467 kb)

## Data Availability

Data supporting the results can be requested by the senior authors upon request.
