# Supplementary material for: Impact of resection margin status on survival in advanced N stage pancreatic cancer – a multi-institutional analysis
Source: Langenbecks Arch Surg. 2021 Mar 13;406(5):1481–9. doi: 10.1007/s00423-021-02138-4 (PMC8370927; doi:10.1007/s00423-021-02138-4)
Supplement: Supplementary file 1 — (DOCX 27 kb) [file 423_2021_2138_MOESM1_ESM.docx]

**Impact of resection margin status on survival in advanced N stage pancreatic cancer – a multi-institutional analysis**

Christian Teske, MD^1^; Richard Stimpel^1^; Marius Distler, MD^1^; Susanne Merkel, MD^2^; Robert Grützmann, MD^2*^; Louisa Bolm, MD^3^; Ulrich Wellner, MD^3^; Tobias Keck, MD^3*^; Daniela E. Aust, MD^4^; Jürgen Weitz, MD^1^; and Thilo Welsch, MD^1§*^

**Supplementary Data**

**Tables**

**Tab. S1. Comparison of patients with R0 and R1 resection status stratified by lymph node stage**

| **Variable** | **N+R0** | **N+R1** | **p-value** |
| --- | --- | --- | --- |
| Patients (n/%) | 273/ 73.4% | 99/ 26.6% |  |
| Male sex (n/%) | 134/ 49.1% | 54/ 54.5% | 0.415 |
| Median Age (years) [IQR] | 68 [61-75] | 69 [63-74] | 0.755 |
| Median CEA (µg/l) [IQR] | 2.6 [1.5-4.3] | 3 [1.6-5.2] | 0.282 |
| Median CA19-9 (U/ml) [IQR] | 144 [43.4-484.5] | 252.40 [53.8-929.8] | **<0.001** |
| Operation (n/%)   - PPPD - cPD - TP - DP - Completion pancreatectomy - Appleby | 156/ 57.1%  55/ 20.1%  22/ 8,1%  40/ 14.7%  0  0 | 58/ 58.6%  15/ 15.2%  16/ 16,2%  9/ 9.1%  0  1 (1.0%) | 0.896  0.348  **0.037**  0.219  N/A  0.596 |
| Neoadjuvant therapy (n/%) | 32/ 11.7% | 5/ 5.1% | 0.088 |
| Adjuvant therapy (n/%) | 150/ 54.9% | 62/ 62.6% | 0.125 |
| PV resection (n/%) | 85/ 31.1% | 40/ 40.4% | 0.122 |
| Arterial resection (n/%) | 18/ 6.6% | 5/ 5.1% | 0.762 |
| Median blood loss (ml) [IQR] | 650 [400-1175] | 1000 [500-1850] | 0.057 |
| Complications ≥ grade 3, (n/%) | 57/ 20.9 % | 24/24.2% | 0.581 |
| POPF grade B-C (n/%) | 24/ 8.8% | 3/ 3.0% | 0.096 |
| Median hospital stay (d) [IQR] | 16 [12–22.75] | 17 [13–25] | 0.411 |
| Postoperative hemorrhage (n/%) | 24/ 8.8% | 12/ 12.1% | 0.446 |

cPD, classical pancreatoduodenectomy; DP, distal pancreatectomy; IQR, interquartile range; N+, corresponding to TNM stages pN1 and pN2; PPPD, pylorus-preserving pancreatoduodenectomy; PV, portal vein; POPF, postoperative pancreatic fistula; TP, total pancreatectomy

**Tab. S2. N-status dependent univariate Cox regression analysis**

| **Variable** | **p-value** | **Hazard ratio** |
| --- | --- | --- |
| pN0; R1 | **<0.001** | 3.07 |
| pN1; R1 | 0.066 | **–** |
| pN2; R1 | 0.314 | **–** |

**Tab. S3. Characterization of the subgroup of patients with neoadjuvant treatment (n=95)**

| **Variable** | **Value** | **%** |
| --- | --- | --- |
| Median Age (years) [IQR] | 64 [55-69] | - |
| Male Sex (n) | 47 | 49.5 |
| Overall survival (months) [IQR] | 19 [16.3-24.2] | - |
| R0 resection status (n) | 79 | 83.2 |
| Median hospital stay (d) [IQR] | 16 [12-27.25] | - |
| POPF grade B-C (n) | 3 | 3.2 |
| Complications ≥ grade 3 (n)* | 20 | 21.1 |

* According to the Clavien-Dindo classification of complications

**Tab. S4. N-status dependent univariate Cox regression analysis (including operations from 2011-2018)**

| **Variable** | **p-value** | **Hazard ratio** |
| --- | --- | --- |
| pN0; R1 | **<0.001** | 2.67 |
| pN1; R1 | 0.759 | **–** |
| pN2; R1 | 0.126 | **–** |

**Figure Legend**

**Fig. S1 Survival analysis of adjuvant treatment**

Kaplan-Meier survival curves were calculated for adjuvantly treated patients with respect to the lymph node stage. Neoadjuvant treatments were excluded. Plots represent pN0 (A, n=88, *P*<0.001), pN1 (B, n=122, *P*<0.01) and pN2 (C, n=73, *P*=0.8). pN2 patients had no survival benefit with R0 resection compared to R1 resected cases.

**Fig. S2 Survival analysis of neoadjuvant treatment**

Kaplan-Meier survival curves were calculated for neoadjuvant therapy with respect to lymph node stage. Plots represent the entire study cohort (A, n=620) as well as pN0 (B, n=233), pN1 (C, n=241) and pN2 (D, n=146) patients. Significant differences in survival curves were obtained for pN1 patients only (*P*=0.03).
