# Supplementary figures and images for: Impact of resection margin status on survival in advanced N stage pancreatic cancer – a multi-institutional analysis
Source: Langenbecks Arch Surg. 2021 Mar 13;406(5):1481–9. doi: 10.1007/s00423-021-02138-4 (PMC8370927; doi:10.1007/s00423-021-02138-4)

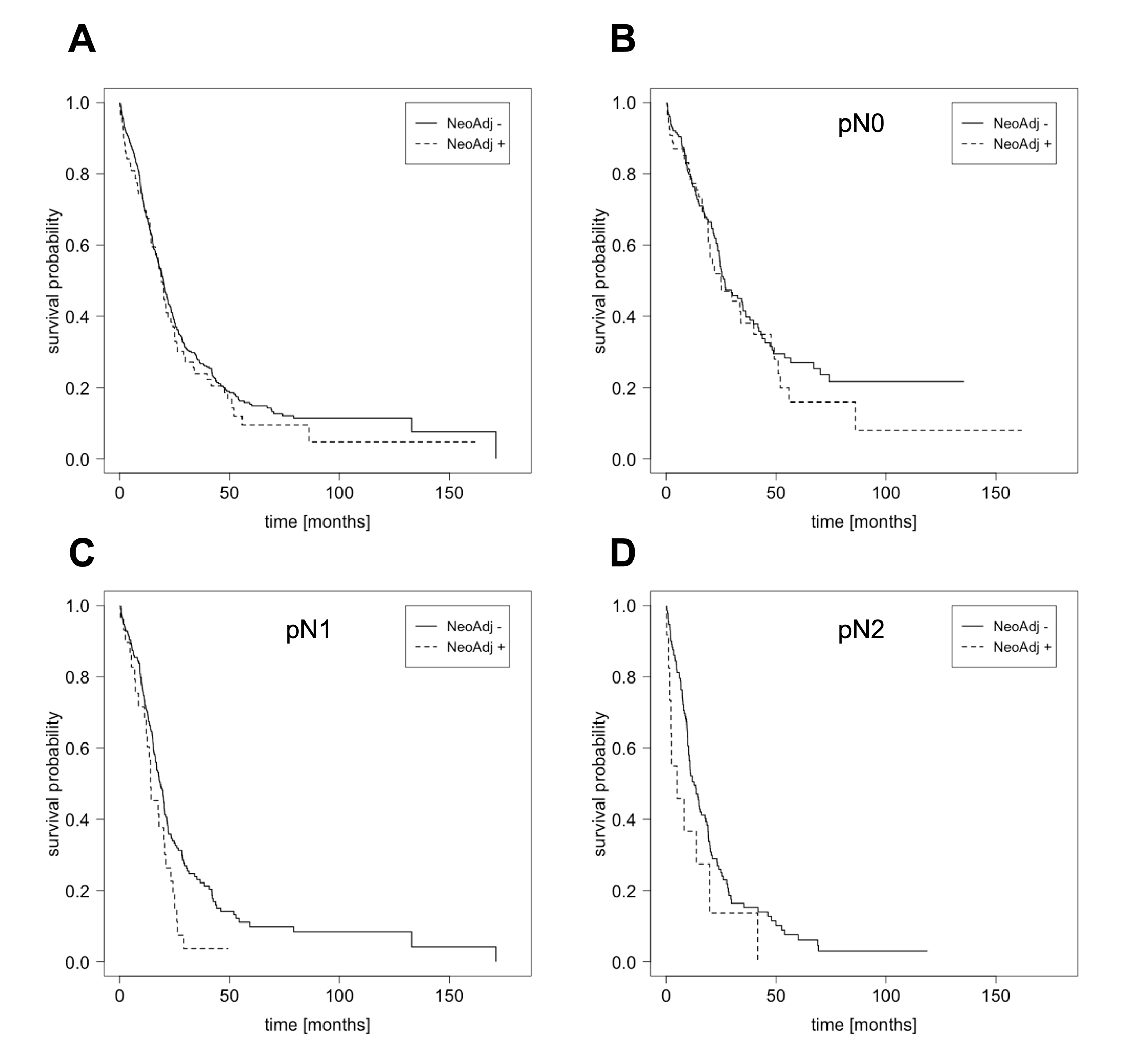

Supplement: Supplementary file 2 — (PNG 371 kb) [file 423_2021_2138_Fig4_ESM.png]

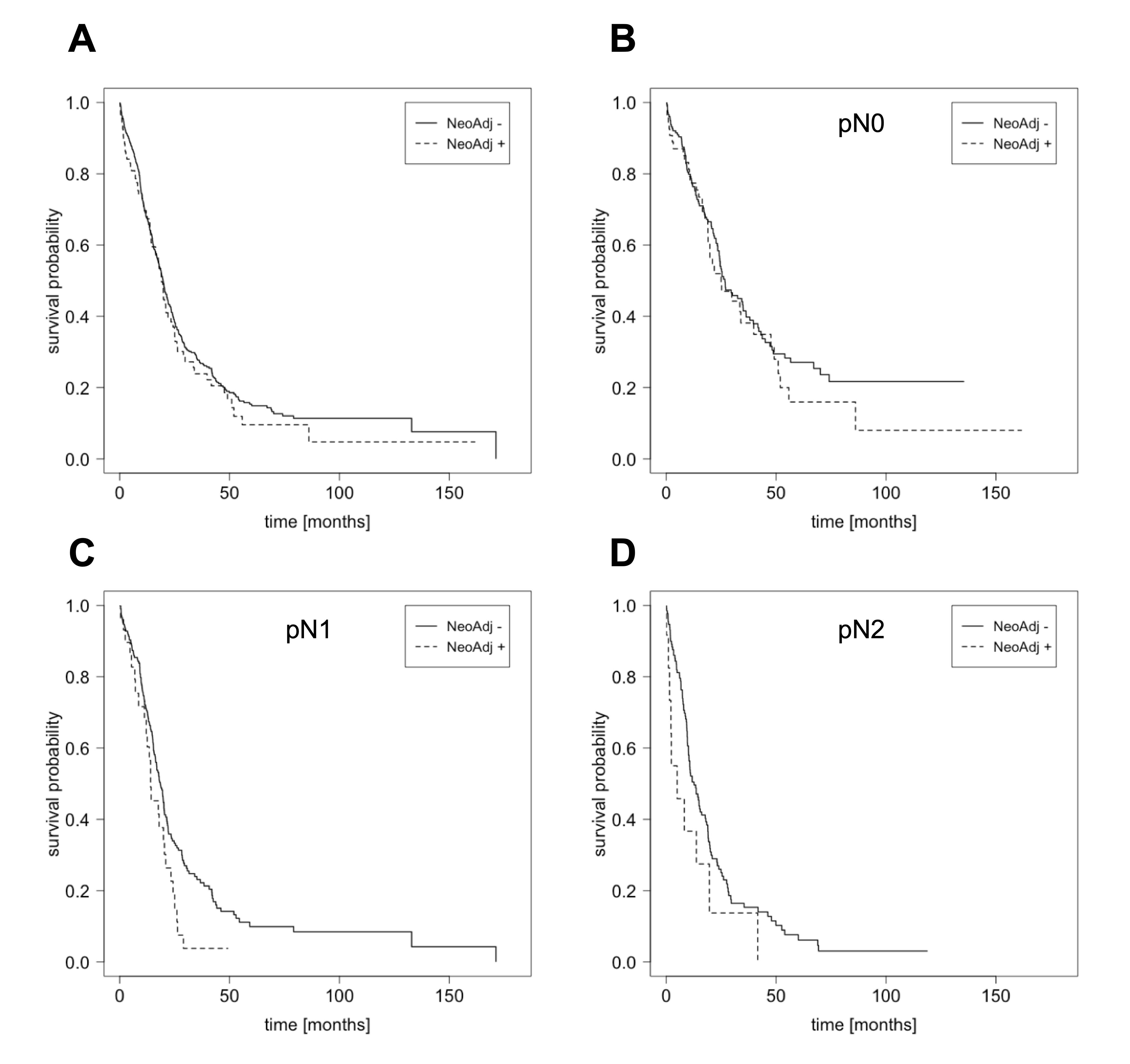

Supplement: Supplementary file 3 — High resolution image (TIFF 4528 kb) [file 423_2021_2138_MOESM2_ESM.tiff]

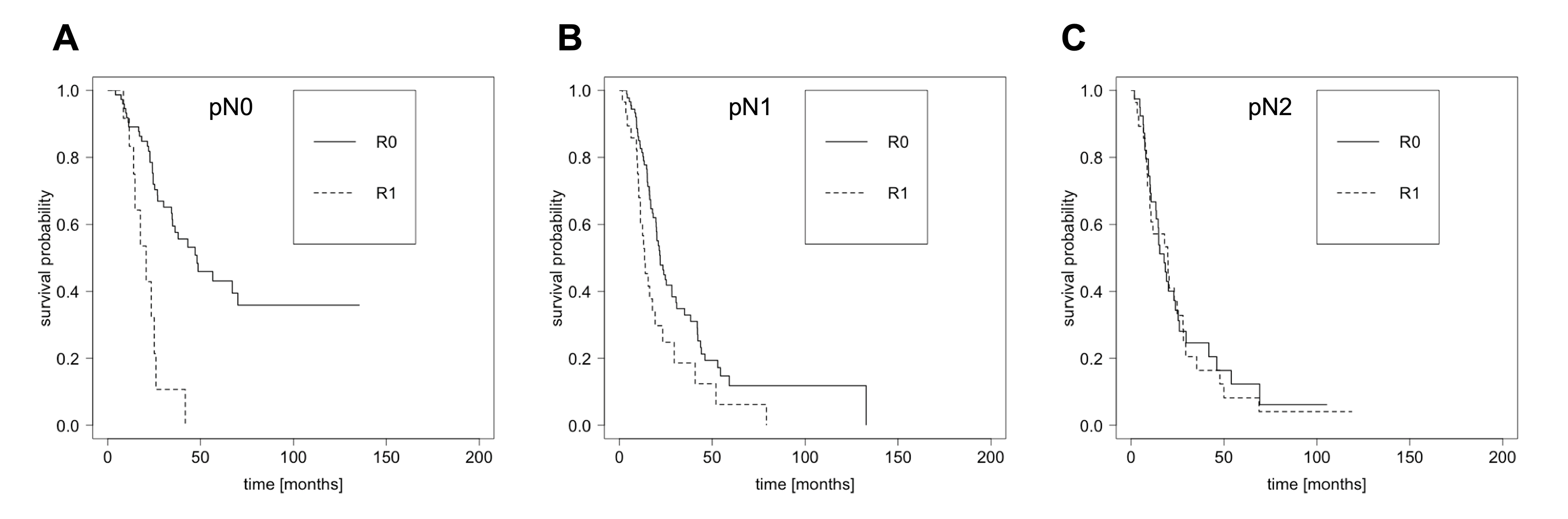

Supplement: Supplementary file 4 — (PNG 253 kb) [file 423_2021_2138_Fig5_ESM.png]
